# Comparison of child morbidity in regions of Ostrava, Czech Republic, with different degrees of pollution: a retrospective cohort study

**DOI:** 10.1186/1476-069X-12-74

**Published:** 2013-09-03

**Authors:** Miroslav Dostal, Anna Pastorkova, Stepan Rychlik, Eva Rychlikova, Vlasta Svecova, Eva Schallerova, Radim J Sram

**Affiliations:** 1 Institute of Experimental Medicine AS CR, Prague, Czech Republic; 2 Czech Hydrometeorological Institute, Prague, Czech Republic; 3 Public Health Institute in Usti nad Labem, Usti nad Labem, Czech Republic; 4 Hviezdoslavova 18, Ostrava, Czech Republic

**Keywords:** Ostrava, Children, Age-specific morbidity, Incidence, ICD-10, Wheezing, Benzo[a]pyrene, PM_10_

## Abstract

**Background:**

To confirm or refute the hypothesis that the morbidity of children (since birth to age 5) born and living in the heavily polluted (PM_10_, benzo[a]pyrene) eastern part of Ostrava, Czech Republic, was higher than the morbidity of children living in other parts of the city.

**Methods:**

Ten pediatricians in 5 districts of Ostrava abstracted the medical records of 1878 children born in 2001–2004 to list all illnesses of each child in ICD-10 codes. The children were divided into four groups according to their residence at birth and thereafter. Most of the children in the eastern area were living in the city district Radvanice and Bartovice.

**Results:**

We report on the incidence of acute illnesses in 1535 children of Czech ethnicity in the first 5 years of life. The most frequent acute illnesses (over 45% of all diagnoses) were upper respiratory infections (URI: J00-J02, J06). In the first year of life, the incidence of URI in 183 children in the eastern area – 372 illnesses/100 children/year – was more than twice as high as in the other 3 areas with a total number of 1352 children. From birth to the age of 5 years, the incidences of pneumonia, tonsillitis, viral infections (ICD-10 code B34) and intestinal infectious diseases were also several times higher in children living in the eastern part of Ostrava. The lowest morbidity was found in children living in the less polluted western part of the city.

**Conclusions:**

The children born and living in the eastern part of the city of Ostrava had from birth through 5 years significantly higher incidence rates of acute illnesses than children in other parts of Ostrava. They also had a higher prevalence of wheezing, atopic dermatitis and allergic rhinitis.

## Background

**Figure 1:**
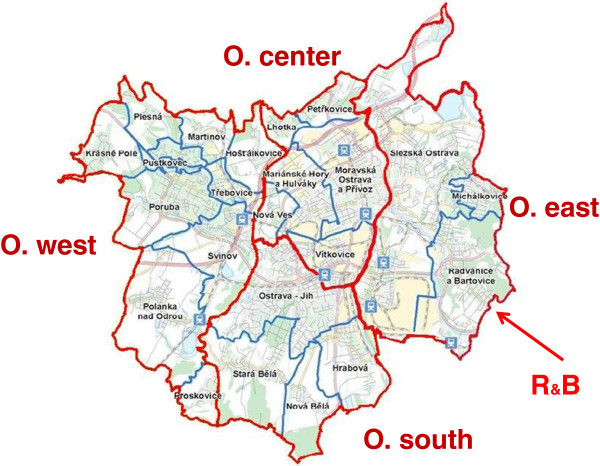
The four regions of the city of Ostrava.

The city of Ostrava is the largest industrial city in the Moravian-Silesian region (MSR) of the Czech Republic. This region is bordered on the west, east and partially also on the south by mountains, forming a basin with frequent winter temperature inversions. The region is a heavily polluted industrial area. Concentrations of PM_10_, PM_2.5_, and benzo[a]pyrene (B[a]P) significantly exceed limit values [1]. The most important industrial sources of air pollution are metallurgy, steel plants, coke ovens and power generating facilities [2]. The population density in the MSR is also associated with high-intensity local vehicular transport and local heating. Almost fifty percent of the MSR inhabitants use central heating, 34% natural gas, 10% coal, 3% electricity, and 3% wood [3]. The prevailing wind direction in Ostrava is SW (especially in winter), with NE winds being the second most frequent. The episodes of highest immissions depend on the wind direction and speed, locality and season [4]. A contribution of sources located on Polish territory to the air pollution load in the city of Ostrava cannot be excluded [5]. Among the extremely polluted areas is the Ostrava city district Radvanice and Bartovice (R&B, Figure 1). One part of the research project Airgen aimed at analyzing the morbidity of children in Ostrava to confirm the hypothesis that children living in a polluted environment in R&B suffer more frequently from acute respiratory infections than children living in cleaner parts of Ostrava. We have compared the incidence rates of respiratory infections and other acute illnesses as well as the prevalence of allergic diseases in preschool children registered at 10 pediatric offices in 5 districts of the city of Ostrava.

## Methods

### Ostrava study

In 2009 and 2010 a total of 1887 children born in 2001–2004 and registered with 10 pediatric offices in 5 districts of the city of Ostrava took part in the study. An informed consent signed by the parents was approved by the Ethical Committee of the Institute of Experimental Medicine of the Czech Academy of Sciences. Mothers were asked to complete a written questionnaire on the home environment of the child, including indoor air pollution sources and other factors potentially related to respiratory illnesses, i.e., breastfeeding, day-care, the number of older siblings, the number of smokers in the household, the type of flat, heating of the flat, family history of allergies, keeping pets, mold and dampness in flat etc. We have also asked mothers to give pediatricians an informed parental permission to inform us about the present health status of children and all illnesses they suffeered from birth to the age of 6. We have not done any further follow-up or collected any biological samples.

The pediatric questionnaire included information about delivery, breastfeeding, congenital defects, serious chronic illnesses, allergies and the results of skin testing. The pediatricians abstracted their medical documentation to list in the codes of International Classification of Diseases, Tenth Revision (ICD-10) all illnesses of the child from birth to the age of 6 years. For the purpose of analysis, the diagnoses were integrated into broader groups (Table 1).

**Table 1:** The major disease groups in children of Czech ethnicity from birth to age of 5

**Group of diseases**	**ICD-10 codes**	**Frequency of dg**	**% of total**
Pneumonia	J12, J14-16, J18	202	0.7
Otitis media	H65-67, H92	961	3.5
Intestinal infectious diseases	A02-04, A08-09	1,274	4.7
Acute laryngitis and tracheitis	J04	1,722	6.3
Viral illnesses	B15, B18, B24-27, B34	1,898	6.9
Acute tonsillitis	J03	2,092	7.6
Acute bronchitis	J20-21, J40	2,214	8.1
Upper respiratory infections	J00-J02, J06	12,964	47.4
All diagnoses of acute illnesses		27,368	100.0

### Geographical stratification according to the levels of ambient pollution

In 1997 Williams et al. [6] followed the exposure of adults to benzo[a]pyrene in Ostrava using both personal and stationary monitors. To this end they divided Ostrava into three regions according to previous studies that mapped pollution by organics. The first region consisted of the western part of the city, while the eastern part was divided horizontally (approximately in the middle) into northeastern and southeastern regions. Because of high levels of PM_10_ and benzo[a]pyrene monitored in recent years in the eastern part of the city of Ostrava, we have further divided the northeastern region and thus we distinguish four Ostrava regions: west, center, east and south (Figure 1). The yearly means of PM_10_ for each locality of residence were calculated using GIS and the data from surrounding monitoring stations in Ostrava, also taking into consideration the prevailing wind direction. The concentrations of PM_10_ were determined by two-dimensional interpolation of data from the nearest monitoring stations. The total means of PM_10_ for the four Ostrava regions were weighted by the number of children taking part in our study in each locality (Table 2). The yearly means of B[a]P are values measured by stationary monitoring stations in O. east, west and center. There are no data for O. south (Table 2).

**Table 2:** **Weighted yearly means of PM**_**10 **_**and the yearly means of benzo[a]pyrene**

	**2001**	**2002**	**2003**	**2004**	**2005**	**2006**	**2007**	**2008**	**2009**
**PM10**	**μg/m3**	**μg/m3**	**μg/m3**	**μg/m3**	**μg/m3**	**μg/m3**	**μg/m3**	**μg/m3**	**μg/m3**
Ostrava south	47.3	44.9	50.8	43.7	48.6	43.9	37.6	37.0	39.9
Ostrava east	46.6	50.8	53.3	40.3	53.4	60.9	59.8	47.8	47.0
Ostrava center	44.2	49.8	54.7	43.3	50.7	50.4	44.1	43.2	42.6
Ostrava west	33.9	40.1	45.8	27.7	31.8	40.1	24.2	26.2	28.7
**B[a]P**	**ng/m3**	**ng/m3**	**ng/m3**	**ng/m3**	**ng/m3**	**ng/m3**	**ng/m3**	**ng/m3**	**ng/m3**
Ostrava east	-	-	7.9	10.0	10.3	11.7	8.9	9.3	9.2
Ostrava center PZ	-	-	7.8	6.5	9.2	6.8	6.4	5.1	5.5
Ostrava center MH	-	-	7.2	4.3	4.5	4.9	4.1	3.9	4.8
Ostrava west	-	-	-	2.1	3.2	3.7	2.2	3.5	3.3

B[a]P was monitored at 4 stations: O. center PZ = Privoz, O. center MH = Marianske Hory (see Figure 1).

### Children

In the Czech Republic each child has to be registered with a pediatrician. In 8 out of 10 participating pediatric offices, more than 90% of registered children (1583 children) took part in the study. In the remaining two offices 69.7% and 74.1% of registered children participated (304 children). In total 1887 children took part in the study. Subsequently applied exclusion criteria included being born outside Ostrava (92 children), birth weight <1500 grams (54 children), congenital defects, tumors, cerebral palsy and long-term preventive administration of antibiotics (27 children), incomplete medical documentation (5 children) and a stay in a children’s home (1 child). The present report is focused on children of Czech ethnicity since their lifestyle and proneness to allergies differ from those of Roma children [7]. The analysis of morbidity was also limited to children born in at least the 36th week of gestation and still living in the same region of Ostrava where they were born. The follow-up of children who spent in the current year less than 11 month at the original address (i.e., region of Ostrava) was discontinued. Therefore, the number of children in the present study decreased from 1535 in the first year of life to 1404 in the 5th year (Table 3). We do not report on the morbidity of children in the 6th year of life because we do not have the data for children born in 2004. The diagnoses of particular illnesses in ICD-10 codes were merged into broader groups (Table 1). The group of upper respiratory infections (URI) included codes for the diagnoses acute nasopharyngitis (common cold, J00), acute pharyngitis (J02) and acute upper respiratory infections of multiple and unspecified sites (J06).

**Table 3:** Numbers of children included in the present analysis stratified according to their residence and age

	**1st year**	**2nd year**	**3rd year**	**4th year**	**5th year**
Ostrava south	708	698	687	682	677
Ostrava east	183	176	174	172	170
Ostrava center	348	326	315	298	289
Ostrava west	296	288	280	272	268
Total	1535	1488	1456	1424	1404

### Statistics

The Kruskal-Wallis equality-of-populations rank test was used for bivariate analyses. Given that the frequencies of most illnesses in individual children were extremely variable, negative binomial regression, suitable for overspread variables, was used for multivariate analyses of the associations of the regions of residence with the incidence rates of respiratory illnesses. The significance of associations was determined by the rate ratio (RR), statistically significant probability and a 95% confidence interval not including the value 1. Confounders were determined by changes in the regression coefficient of more than 15% after removal of the variable. All tests were performed using STATA 12 software.

## Results

### Ambient air pollution

The yearly means of PM_10_ show that the children born and living in the region O. west were exposed to the lowest immissions of PM_10_ (Table 2) as only in 2003 did the yearly mean exceed 40.1 μg/m^3^. In 2001–2003 there was practically no difference between the rather steep increases in the yearly means of PM_10_ in the other three regions of Ostrava. After a decrease in 2004 the PM_10_ levels started to increase again and peaked in 2006 and 2007. In this period the levels of PM_10_ in the four Ostrava regions noticeably differed, and the levels of PM_10_ increased in the order O. west, south, center and east. In the latter region the mean concentration of PM_10_ in 2006 reached 61 μg/m^3^. The yearly means of benzo[a]pyrene are available for the period from 2003 to 2011. The lowest yearly means in the range 2.1 to 3.7 ng/m^3^ of B[a]P were observed in O. west. The highest levels in the range 7.9 to 11.7 ng/m^3^ of B[a]P were recorded by the monitoring station in R&B in the region O. east. From 2003 to 2006 the yearly means of B[a]P in O. east increased by 48%. The 6 months means of the winter and autumn months ranged from 12.2 to 18.7 ng/m^3^ in O. east (data not shown). B[a]P was not monitored in O. south.

### Age- and region-specific morbidity of children

**Figure 2:**
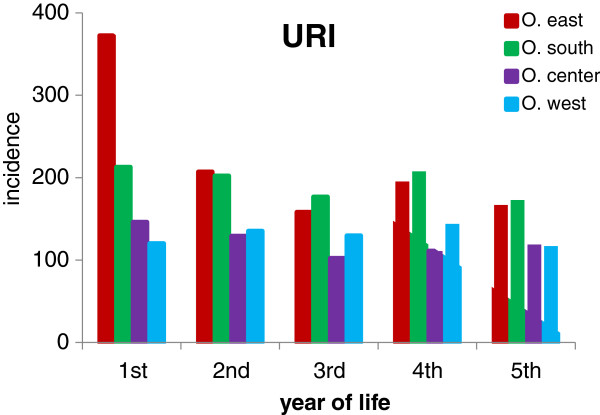
**Incidence of upper respiratory infections (URI) per 100 children per year of life.** Numbers of children stratified by age and region of residence are presented in Table 3.

**Figure 3:**
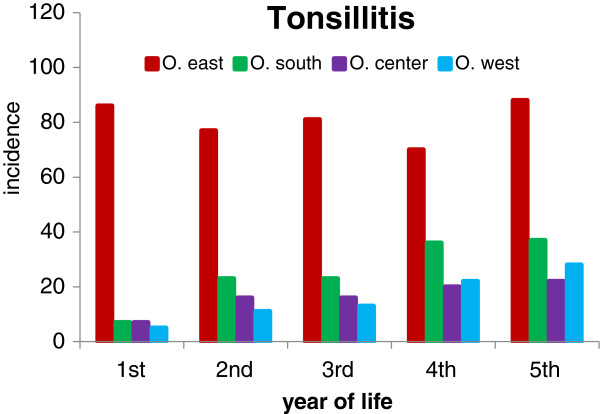
**Incidence of tonsillitis per 100 children per year of life.** Numbers of children stratified by age and region of residence are presented in Table 3.

**Figure 4:**
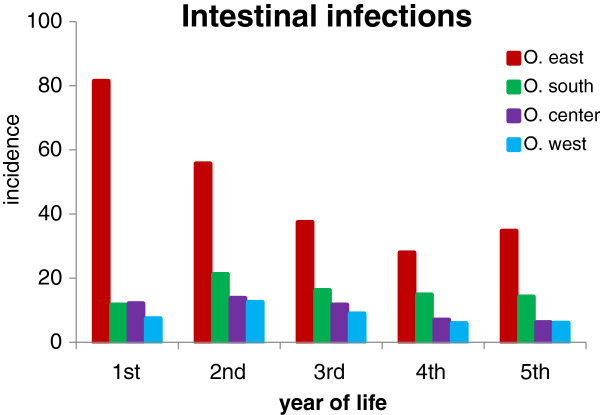
**Incidence of intestinal infectious diseases per 100 children per year of life.** Numbers of children stratified by age and region of residence are presented in Table 3.

Acute URI represented 47.4% of the total number of 27,368 diagnoses of acute illnesses evaluated in our study (Table 1). Their age-specific incidence rates/100 children/year in the four Ostrava regions are shown in Figure 2. The highest incidence - 372 episodes per 100 children in the first year of life - was found in the children from the region O. east. In the second year of life the incidence rate of URI in O. east decreased by about 50% to the level of the incidence of URI in O. south, and this similarity persisted to the 5th year of life. The incidence rates of URI in children living in O. center and O. west were remarkably lower. The incidence rate of tonsillitis in the 1st year of life of children born and living in O. east (86/100 children/year) was approximately 8 times higher than in the other regions of Ostrava. It remained at the same level till the 5th year of life (Figure 3). The incidence rate of tonsillitis in the other regions of Ostrava increased with age to a maximum of 37/100 children in the 5th year of life in O. south. The incidence rate of intestinal infectious diseases was highest in the O. east children with a rate of 81/100 children/year in the 1st year, decreasing to 27/100 children/year in the 3rd year (Figure 4). The incidence rates in the other 3 regions remained in the range from 6 to 35/100 children/year; the incidence plots for O. center and O. west were almost identical.

**Figure 5:**
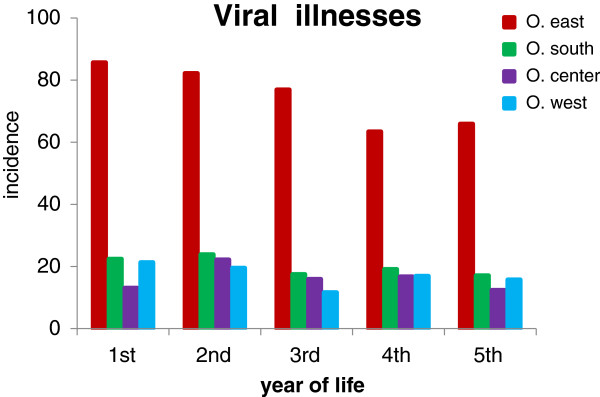
**Incidence of viral illnesses per 100 children per year of life.** The vast majority of diagnoses in O. east were infections of unspecified site (ICD code B34). Numbers of children stratified by age and region of residence are presented in Table 3.

The children in O. east also had the highest incidence rate of viral illnesses, namely of unspecified viral infections (ICD code B34). The rate of 86/100 children/year in the 1st year decreased rather slowly to 63/100 children in the 4th year (Figure 5). The incidence rates in children in the other three regions were similar, ranging from 12 to 24/100 children/year.

**Figure 6:**
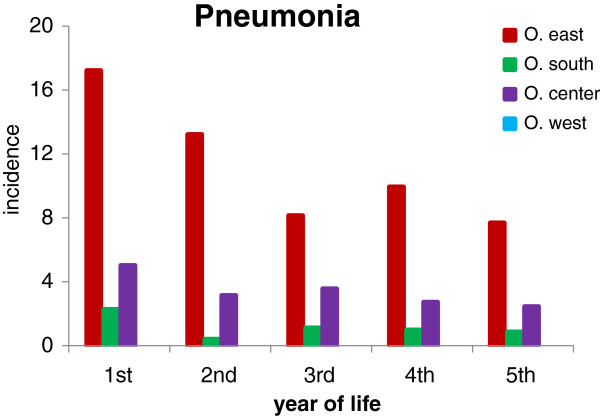
**Incidence of pneumonia per 100 children per year of life.** Numbers of children stratified by age and region of residence are presented in Table 3. In O. west there was only 1 case of pneumonia in the 1st and another one in the 5th year.

Finally, the incidence rate of pneumonia was higher in children born and living in O. east than in the other regions. It decreased from 17/100 in the 1st year to 8/100 in the 3rd year and remained approximately at this level in the 4th and 5th year (Figure 6). In O. west there was only 1 case of pneumonia in the 1st and another one in the 5th year.

The incidence of otitis media was quite low in the first three years, then peaked in the 4th year of life in O. south at 24/100 children/year. The peak incidence was also reached in the 4th year in O. west and east and in the 5th year in O. center. The plots of the age incidence of laryngitis and tracheitis show two distinctly different pairings. Children in O. west and O. east had a lower incidence with a plateau from the 3rd to the 5th year of age (about 15 diagnoses/100 children/year). The other two plots (O. south and O. center) had their highest values at about 35 diagnoses/100 children/year in the 4th and 5th year of age. The incidence plots of bronchitis peak in the 4th or the 5th year of age. The highest yearly incidence from the 2nd to the 4th year was found in children living in O. center.

### Covariates

Sociodemographics, the indoor environment and other characteristics stratified by region of residence are presented in Table 4. There were several differences between families living in O. east and in the other three regions. More mothers in O. east attained the lowest level of education and fewer mothers attained the highest levels. More mothers smoked during pregnancy and fewer children were breastfed and attended day care. The biggest differences were in the type of housing, since in R&B (where most children in our sample from O. east lived) family houses prevail over apartment block flats and, consequently, space heating (gas, coal/wood) and mold in flats were more frequent and gas appliances in flats were less frequent. Also, more children had siblings under 14 with allergies. However, none of these covariates confounded associations of the increased incidence of acute infections in children born and living in the O. east region.

**Table 4:** Data from the maternal questionnaires

**Variables**		**O. south**	**O. east**	**O. center**	**O. west**	**P. (KW)**
		**%**	**%**	**%**	**%**	
Gender	Boys	49.7	49.2	55.2	48.3	
	Girls	50.3	50.8	44.8	51.7	
Delivery	Sectio Caesarea	17.4	16.4	19.4	18.7	
	Phototherapy*	5.5	1.6	7.2	4.4	= 0.028
Birth weight	Mean (grams)	3380	3380	3380	3399	
	SD (grams)	478	492	481	455	
Age of mothers at delivery	<20 years	4.2	5.5	6.0	3.4	
	20 to 30 years	64.7	68.9	67.0	67.8	
	>30 years	31.0	25.7	27.0	28.8	
Mother’s education	Low	9.1	16.4	10.1	5.1	
	Middle	40.2	38.3	35.1	29.7	
	High	50.7	45.4	54.9	65.2	
Breastfeeding	Breast fed	92.8	86.3	93.7	92.9	= 0.009
	Months – mean	8.7	7.6	8.8	11.0	
	Months – SD	9.1	8.1	9.1	20.9	
Smoking of mothers	During pregnancy	12.0	17.5	18.1	9.1	
	During the study	31.5	32.2	35.6	23.6	= 0.019
Exposure to ETS*	Any adult	48.6	54.6	49.9	42.7	
Type of flat	Family house	11.0	55.0	3.0	15.5	< 0.001
(in the 1st year)	Brick block flats	23.6	36.8	36.0	36.1	
	Concrete block flats	65.5	8.2	61.0	48.5	
Type of heating	Distant	65.5	8.2	61.0	48.5	< 0.001
	Gas	16.0	48.3	15.4	13.1	
	Coal/wood	2.8	30.2	2.1	2.8	
Mold in flat	Yes	24.2	36.8	12.9	18.9	< 0.001
Gas appliances in flat	Yes	86.6	66.3	90.8	89.8	< 0.001
Other children under 14	Yes	51.5	55.7	48.9	41.2	
Family history of allergies	Mother	37.2	44.8	34.2	33.1	
	Father	21.1	23.0	25.0	28.7	
	Siblings	20.9	31.1	26.1	26.4	= 0.016
Pets	Cat	6.4	9.8	5.2	4.7	
	Dog	20.9	19.1	17.5	20.9	
	Birds	7.2	8.2	6.0	7.8	
	Other*	23.6	20.8	21.8	22.0	
Day care	3rd and 4th year	56.8	40.4	52.7	63.5	< 0.001
	4th and 5th year	89.3	72.9	89.9	92.9	< 0.001

Categorical covariates except birth weight and the length of breastfeeding. KW-Kruskal Wallis test.

*Phototherapy - neonatal icterus treated with phototherapy, *ETS - environmental tobacco smoke.

*Other - rabbits, guinea pigs, hamsters, degus etc.

### Incidence rate ratios

Differences between the incidence rates of illnesses in children living in the four regions of Ostrava were evaluated in two age categories: from birth to the age of 2 years and from the age of 2 to 5 years (Table 5). Associations of the incidence rates of illnesses with four level region variables were tested using negative binomial regression. As the basic level we chose residence in O. east (Table 6). In both age categories the incidence rates of bronchitis and otitis media did not differ, while the rates of tonsillitis, viral illnesses, and intestinal infections in the regions O. south, west and center were significantly lower than in O. east. The rate ratios ranged from 0.51 to 0.11. The incidence rates of URI were significantly higher than in the remaining regions in the age category of 0–2 years, whereas in the category 2–5 years there was no difference between the O. east and O. south. The incidence of pneumonia was significantly higher in the age category 0–2 years and too low to be tested in the older group. The incidence of laryngitis and tracheitis in O. east varied with age; in the older category it was lower in O. east than in O. south and O. center. The RRs were adjusted for gender, type of delivery, neonatal icterus treated with phototherapy, year of birth, breastfeeding, maternal age and education, atopic dermatitis, smoking of the mother, exposure to environmental tobacco smoke, having an older sibling, allergy of an older sibling, type of flat (1st year), households with a gas appliance and mold in the flat in the 1st year of life. The multivariate regressions for the age category 2–5 years were also adjusted for recurrent wheezing, allergic rhinitis and attending day-care in the 4th and the 5th year of life.

**Table 5:** Cumulative incidence rates of selected illnesses per 100 children of Czech ethnicity

	**Age 0–2 years**				**Age 2–5 years**			
**Ostrava**	**East**	**South**	**Center**	**West**	**East**	**South**	**Center**	**West**
**Number of children**	**N = 176**	**N = 698**	**N = 326**	**N = 288**	**N = 170**	**N = 677**	**N = 289**	**N = 268**
URI	584.7	419.5	276.7	253.1	520.6	559.7	328.7	392.2
Bronchitis	56.3	60.9	56.1	43.8	88.8	81.2	104.2	89.2
Tonsillitis	162.5	29.5	23.6	16.3	239.4	94.1	59.2	64.6
Viral illnesses	166.5	47.4	35.9	41.7	205.9	53.5	47.1	43.7
Laryngitis/tracheitis	37.5	33.4	38.7	19.4	50.0	93.4	100.0	47.4
Intestinal infections	139.8	32.5	25.8	19.8	101.2	45.3	23.5	20.5
Otitis media	18.2	16.0	18.7	15.3	41.8	56.9	35.6	39.2
Pneumonia	29.5	2.9	8.0	0.3	25.9	3.1	9.0	0.4
Total	1194.9	642.1	483.4	409.7	1273.5	987.1	707.3	697.0

**Table 6:** Rate ratios (RR) Ostrava east/other regions

	**O. south**			**O. center**			**O. west**		
**Age 0 to 2 years**	**RR**	**P**	**95% CI**	**RR**	**P**	**95% CI**	**RR**	**P**	**95% CI**
URI	0.69	<0.0001	0.61 – 0.79	0.45	<0.0001	0.39 – 0.52	0.44	<0.0001	0.38 – 0.52
Bronchitis	-	NS	-	-	NS	-	-	NS	-
Tonsillitis	0.19	<0.0001	0.14 – 0.25	0.16	<0.0001	0.11 – 0.22	0.11	<0.0001	0.08 – 0.16
Viral illnesses	0.30	<0.0001	0.28 – 0.38	0.23	<0.0001	0.17 – 0.30	0.27	<0.0001	0.21 – 0.35
Laryngitis/tracheitis	-	NS	-	-	NS	-	0.53	0.005	0.34 – 0.83
Intestinal infections	0.33	<0.0001	0.25 – 0.43	0.25	<0.0001	0.18 – 0.35	0.18	<0.0001	0.14 – 0.28
Otitis media	-	NS	-	-	NS	-	-	NS	-
Pneumonia	0.1	0.001	0.05 – 0.22	0.31	0.001	0.15 – 0.64	-	NT	-
**Age 2–5 years**	**RR**	**P**	**95% CI**	**RR**	**P**	**95% CI**	**RR**	**P**	**95% CI**
URI	-	NS	-	0.62	<0.0001	0.53 – 0.73	0.76	<0.0001	0.65 – 0.88
Bronchitis	-	NS	-	-	NS	-	-	NS	-
Tonsillitis	0.40	<0.0001	0.32 – 0.50	0.24	<0.0001	0.18 – 0.32	0.27	<0.0001	0.20 – 0.35
Viral illnesses	0.28	<0.0001	0.22 – 0.35	0.24	<0.0001	0.18 – 0.31	0.22	<0.0001	0.17 – 0.29
Laryngitis/tracheitis	1.88	<0.0001	1.33 – 2.65	1.98	<0.0001	1.36 – 2.89	-	NS	-
Intestinal infections	0.51	<0.0001	0.38 – 0.69	0.24	<0.0001	0.16 – 0.35	0.22	<0.0001	0.15 – 0.33
Otitis media	-	NS	-	-	NS	-	-	NS	-
Pneumonia	-	NS	-	-	NS	-	-	NS	-

*CI*, Confidence interval; *NS*, P > 0.05, *NT*, Not tested. Negative binomial regression, 1444 children living in the region where they were born up to the age of 2 or 5 years.

The RRs were adjusted for gender, type of delivery, neonatal icterus treated with phototherapy, year of birth, breastfeeding, maternal age and education, atopic dermatitis, smoking of the mother, exposure to environmental tobacco smoke, having an older sibling, allergy of an older sibling, type of flat (1st year), households with a gas appliance and mold in the flat in the 1st year of life, and in the older age also for wheezing and allergic rhinitis.

### Prevalence of allergies

The prevalence of allergies was evaluated in 1404 children of Czech ethnicity, born and living in the city of Ostrava till the age of 5 years, as children of Roma ethnicity have allergies rather seldom [7]. The overall prevalence of recurrent wheezing (WH) was 14.0%, and the prevalence of allergic rhinitis (AR) and atopic eczema (AE) were 10.8% and 17.8%, respectively. The allergies were diagnosed by pediatricians and confirmed by allergists. Only 7 children with reported recurrent wheezing did not see an allergist. The prevalence of all three diagnoses was the highest in children born and living in the region O. east: WH 37%, AE 35% and AR 24%. Also, combinations of two or three types of allergy were most frequent in O. east: 37% of children with WH had also AE, while 57% of children with WH were also diagnosed with AR. Finally, 43% of children with WH were diagnosed with all three allergies (Table 7). Skin tests to aeroallergens were applied to most children with a suspected allergy. The highest percentages of children in all four regions of Ostrava were sensitised to pollen and mites. The children living in O. east were sensitised less frequently than children in the other parts of Ostrava (Table 7). However, the panels of tests applied were not necessarily identical.

**Table 7:** Prevalence of wheezing and allergies among children of Czech ethnicity stratified by region of residence

**Region**	**Number and % of children**				**% of children with wheezing**				**Skin tests, number of tested, % positive**					
	**% of children**													
	**N**	**WH%**	**AE%**	**AR%**	**only WH**	**WH + AR + AE**	**WH + AE**	**WH + AR**^**1**^	**N**	**Molds**	**Dog**	**Cat**	**Mites**	**Pollen**^**2**^
O. south	677	10.0	15.2	9.0	29.4	23.5	29.4	41.2	104	5.8	14.4	14.4	25.0	45.2
O. east	170	37.1	35.3	24.1	6.3	42.9	36.5	57.1	67	7.7	1.0	1.0	9.6	29.8
O. center	289	10.7	13.8	9.3	46.4	3.0	19.4	32.3	76	17.3	4.8	5.8	27.9	35.9
O. west	268	13.1	17.5	8.2	42.9	17.1	28.6	32.6	47	7.7	4.8	3.8	11.5	21.2

Only children living in the four regions of Ostrava from birth to the age of 5 years are included. WH – wheezing, AE – atopic eczema, AR – allergic rhinitis (without differentiating between hay fever and year-round rhinitis). The prevalence of wheezing and allergies is significantly higher in Ostrava east (Kruskal Wallis test, P = 0.0001). Skin tests in children of Czech ethnicity - the percentages of positive tests in the children tested.

^1^without regards to AE, ^**2**^child positive to any species.

## Discussion

The primary aim of the study was to prove or refute the hypothesis that infants, toddlers and preschool children living in the Ostrava district R&B had higher morbidity rates than children living in other parts of Ostrava. In the Czech Republic, each child has to be registered with a pediatrician. Due to the high percentage of participating children registered with 10 pediatricians, our cohort can be considered a representative sample. We have compared the morbidity of the children by quantifying the incidence rates of acute illnesses in the first five years of life. Our results unequivocally prove the high morbidity of children born and living in the region O. east, most of them in the district R&B. These children had a significantly higher incidence of URI than children in O. center and O. west (in all years) and in O. south in the 1st year. Moreover, in all 5 years they had the highest incidence rates of pneumonia, tonsillitis, intestinal infectious diseases and unspecified viral illnesses. They also had the highest prevalence of recurrent wheezing, atopic dermatitis and allergic rhinitis at the age of 5 years. The lowest incidence of most illnesses was seen in children living in O. west. The children in O. east suffered less frequently from laryngitis and tracheitis and bronchitis than children in the other regions of Ostrava. The low incidence of tracheitis and laryngitis in O. east may be explained by the fact that the environment in O. east is much less urban than in O. south and center. An apparent reason for the low incidence of acute bronchitis is the high prevalence of recurrent wheezing (i.e., obstructive bronchitis) and its treatment.

Our previous study on the health of children (birth to age 10) living in the industrial district of Teplice, Czech Republic [8,9], showed that the incidences of all respiratory illnesses were at the levels seen in O. west, center or south. The only exception was the high incidence of laryngitis and tracheitis in children living in the town of Teplice. This is apparently associated with the urban environment, since the incidence of laryngitis and tracheitis in children living in the other parts of the district of Teplice was lower. The early onset of otitis media is considered as typical in many published reports [10,11]. We saw the peak incidence in the 1st and the 2nd year in children living in the district of Teplice. The highest incidence of OM in Ostrava children occurred in the 4th (O. south, east and west) or the 5th year of life (O. center) [12].

In contrast to the Teplice study, the children in Ostrava were seldom diagnosed with influenza.

The four regions of Ostrava differ in the levels of pollution by PAHs and PM_10_. The WHO scientific review [13] stated that in addition to the carcinogenic effects of PAHs (c-PAHs, carcinogenic polycyclic aromatic hydrocarbons), there is new evidence for early biological effects of PAHs exposure in both children and adults, and that there are no reasons for changing the limit value of B[a]P at 1 ng/m^3^. Miller et al. [14] used personal air monitors for 48 h to determine c-PAHs exposure levels (the sum of eight PAHs including B[a]P) of women in the 3rd trimester of pregnancy. Their children at the age 12 months exhibited more frequent coughing and wheezing when their mothers were exposed to c-PAHs during pregnancy and the children were exposed in the postnatal period to environmental tobacco smoke. An association of acute respiratory infections, sore throat and ear infections in the first year of life of children of mothers exposed to c-PAHs in the second trimester was also suggested by the results of a study performed in Krakow, Poland, which is a region bordering MSR and similar in its industrial status and ambient air pollution [15]. The authors explained this observation by the immunotoxic activity of c-PAHs, which impairs fetal development of the immune system and is responsible for the increased susceptibility of newborns and preschool children to respiratory infections. Children living in the region O. south had the second highest incidence rate of URI in the first year and a slightly higher incidence of URI than the children in O. east in the age 2–5. PAHs are not monitored in O. south.

Dejmek et al. [16] observed the effect of increasing concentrations of c-PAHs in polluted air on intrauterine growth retardation and low birth weight. Choi et al. [17] published the results of another study from Krakow. Personal exposure of mothers to B[a]P in the first trimester was 2.11 ng/m^3^ during March-May and 7.21 ng/m^3^ during December-February. This study showed a significant negative impact of c-PAHs on the growth of the fetus during the first trimester. In our study, there were significantly more newborns with a birth weight under 2,500 grams in O. south than in the other regions of Ostrava [18]. PAH-DNA adducts have been detected in cord and maternal blood [19] and placentas [20]. Prenatal exposure to c-PAHs may be associated with altered lymphocyte immunophenotypic distribution in cord blood [21] and changes in cord serum immunoglobulin E levels [22]. When exposed children were followed until school age, it was observed that prenatal exposure to c-PAHs induced behavioral deficits and neuromotoric disturbances [23-26] and increased the prevalence of asthma bronchiale [27].

Biomarkers of genotoxicity in newborns were compared in a cohort of newborns in Prague and the town of Ceske Budejovice in the southern part of the Czech Republic. B[a]P concentrations and DNA adducts and micronuclei in the leucocytes of cord blood were higher in Ceske Budejovice than in Prague. Moreover, logistic regression suggested a possible association of B[a]P exposure in the third trimester with the percentages of micronuclei in cord blood [28]. These results support the view that prenatal exposure to PAHs can induce a developmental dysbalance of the immune system. However, in our study we have no data on personal exposures, and the data from the monitoring station in R&B are available only since 2003 and for O. south they are completely missing.

We have confirmed the highest prevalence of wheezing during the first 5 years of life in children born and living in the district R&B. These children also had the highest prevalence of allergic rhinitis and of atopic eczema, which may indicate a higher probability of developing permanent asthma bronchiale [29]. The two-stage hypothesis of Gern [30] says that environmental exposures interact with genetic factors during the prenatal and postnatal periods to adversely influence the development of innate and adaptive immunity, which in turn increases the risk for allergic sensitization and atopic diseases. In the second stage, immune dysregulation in infancy increases the risk of developing lower respiratory infections that cause airway inflammation and structural changes during a particularly vulnerable period of lung development, leading to an increased risk of asthma by age 7 years. This hypothesis is applicable to the effects of c-PAHs. As far as the mechanisms of the prenatal effects of c-PAHs are concerned, it has been found that during the fetal phases of development, c-PAHs can change the methylation pattern of CpG cytosine in fetal DNA. Methylation of the promotor parts of genes silence their expression. Several studies reported an association of this type of prenatal effect of c-PAHs with the presence of wheezing in children at the age of 5 years. The presently available data on environmental epigenetics of asthma bronchiale in children have been reviewed [31,32]. Maternal exposure to c-PAHs during pregnancy was also associated with the methylation of the promotor of the IFNγ gene, which may be associated with the differentiation of naive CD4+ T lymphocytes into proallergic TH2 helper lymphocytes [33]. The results of studies on the genotoxicity of c-PAHs performed in the Ostrava region in adults and *in vitro* were reviewed by Sram et al. [1].

**Figure 7:**
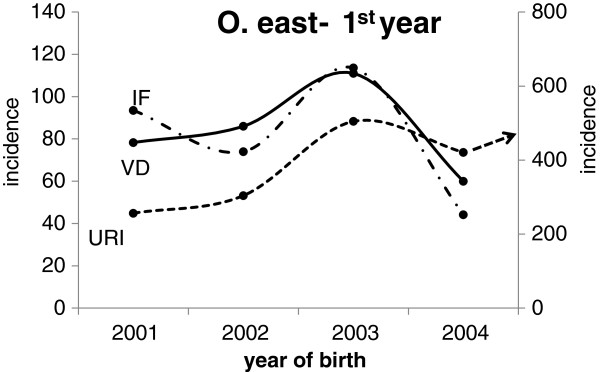
**The incidence (per 100 children/year) of upper respiratory infections (URI, the right *y *axis), viral diseases (left *y *axis) and intestinal infectious diseases (left *y *axis) in the first year of life in infants stratified by the year of birth.**

In the WHO review [13] it is stated that there is increasing evidence for the effects of coarse particles (PM_10-2.5_) on human health, independently of the effects of PM_2.5_. When we stratified the morbidity of O. east children in the 1st year of life according to the year of birth, the highest incidences of URI, intestinal infectious diseases and viral illnesses were found in children born in 2003. On the other hand, the children born in 2004 had the lowest incidences of all illnesses except URI. The incidence rate of URI in the first and second year of life of children born in 2003 and 2004 was significantly higher than in children born in 2001 and 2002, and it was not associated with the season of birth. The incidence plots of URI, viral illnesses and intestinal infectious diseases (Figure 7) resemble the 2001–2004 plots of the yearly means of PM_10_ in Ostrava (see the data in Table 2). From the Czech Hydrometeorological Institute data [34] it follows that this oscillation in the yearly means of PM_10_ was common throughout the Czech Republic and was therefore due to meteorological factors. The time differences in morbidity were limited to children living in O. east and support the view that ambient air pollution affects morbidity in infants.

Our study is a retrospective study. The data were extracted from pediatric documentation. The advantage of this approach is that the records were made at the time of illness and therefore are not memory biased. The disadvantage is, on the other hand, that the effects on morbidity of additional factors, such as children’s way of life (their physical activity, nutrition, etc.) cannot be followed. From the published studies (most of them concerned with schoolers) it follows that there is a connection between life style of children and their parents, the latter of which is dependent on their socioeconomic status e.g., [35], the proxy being mothers’ education. In a systematic review of the studies on unfavorable health outcome related to sedentary behavior of children aged 0–4 years, only low to moderate evidence was reported for the adiposity, psychosocial health and cognitive development [36]. In our study, a high incidence of acute respiratory infections in O. east children was already present in the 1st year of life when the vast majority of children were breastfed. Later on (in the 3rd, the 4th and the 5th year) from 40 to 90% children visited day care, which increased their risk of their being infected (Table 4).

## Conclusions

In comparison with children living in the other regions of Ostrava, the children born and living for 5 years in the region Ostrava east (most of them in the highly polluted district R&B) had: a higher incidence of upper respiratory infections, pneumonia, tonsillitis, intestinal infectious diseases and unspecified viral infections (ICD10 code B34). They also had the highest prevalence of wheezing (37%), atopic dermatitis (35%), allergic rhinitis (24%) and their combinations. Among the four regions of Ostrava, the highest yearly means of PM_10_ and benzo[a]pyrene (in 2006) were recorded at the monitoring station in R&B, whereas the lowest yearly concentrations of both PM_10_ and benzo[a]pyrene occurred in the region Ostrava west with a low morbidity in children. In multivariate models the effects of the region of residence on morbidity in children were not modified by any covariate. Our data are in agreement with the literature data which show that prenatal exposure to benzo[a]pyrene presents a considerable risk to multiple organ systems with negative consequences for postnatal development.

## Abbreviations

AE: Atopic eczema; AR: Allergic rhinitis; B[a]P: Benzo[a]pyrene; ICD-10: International classification of diseases, tenth revision; MSR: Moravian-Silesian region; NE: Northeast; O.: Ostrava; PAHs: Polycyclic aromatic hydrocarbons; c-PAHs: Carcinogenic polycyclic aromatic hydrocarbons; PM10: Particulate matter with an aerodynamic diameter ≤ 10 μm; PM2.5: Particulate matter with an aerodynamic diameter ≤ 2.5 μm; R&B: Radvanice and Bartovice; RR: Rate ratio; SW: Southwest; URI: Upper respiratory infections; WH: Wheezing.

## Competing interests

The authors declare that they have no competing interests.

## Authors’ contributions

MD collected the field data, performed the statistical analysis and drafted the manuscript. AP supervised digitalization of the field data, constructed the final database and took part in performing statistical analysis. VS was responsible for data on pollution of the city of Ostrava. SR and ER calculated the yearly means of PM_10_ for each locality of residence using GIS and the data from surrounding monitoring stations. ES provided advice about composition of questionnaires and analysis of ICD-10 codes. Author RJS conceived the study and participated in its coordination and in drafting the manuscript. All authors have read and approved the final manuscript.
